# An investigation of *NFXL1*, a gene implicated in a study of specific language impairment

**DOI:** 10.1186/s11689-016-9146-9

**Published:** 2016-04-05

**Authors:** Ron Nudel

**Affiliations:** Wellcome Trust Centre for Human Genetics, University of Oxford, Oxford, OX3 7BN UK

**Keywords:** Specific language impairment, NFXL1, Neurogenetics, Neurodevelopment, Language disorder, Transcription factor, Cerebellum

## Abstract

**Background:**

A recent study identified *NFXL1* as a candidate gene for specific language impairment. The protein encoded by this gene is predicted to be a transcription factor based on domain similarities with NFX1, a repressor of HLA class II genes, which have themselves been implicated in specific language impairment. However, there is very little literature on the function of *NFXL1*.

**Methods:**

This report describes a study of *NFXL1* expression in several human tissues and an investigation of differential expression in several specific brain regions through quantitative PCR as well as a study of the protein’s sub-cellular localization in HEK cells and SH-SY5Y cells through immunofluorescence.

**Results:**

The *NFXL1* transcript was found in all investigated tissues. In the brain, a high level of *NFXL1* expression was found in the cerebellum. An analysis of the sub-cellular localization of the protein showed a cytoplasmic pattern in the investigated cells.

**Conclusions:**

The *NFXL1* transcript was present in samples from different tissues; in the brain, a high expression level was found in a region implicated in some language-related pathologies. NFXL1 did not show nuclear localization, suggesting that, if it regulates transcription, certain conditions may be required for it to translocate to the nucleus.

**Electronic supplementary material:**

The online version of this article (doi:10.1186/s11689-016-9146-9) contains supplementary material, which is available to authorized users.

## Background

Specific language impairment (SLI) is diagnosed when a child has problems with language acquisition in the context of otherwise typical cognitive development [1]. Recently, an exome-sequencing study of SLI identified a new candidate gene, *NFXL1* [2]. The protein is predicted to be a transcription factor based on domain similarities with NFX1 (a transcription factor which represses HLA class II genes [3]), which include RING-type and NF-X1-type zinc finger predicted domains. Interestingly, some alleles of HLA class II genes were highlighted in a study of SLI and HLA alleles [4]. To date, *NFXL1* has received little attention, but a study that examined a cloned fragment of the gene in HeLa cells found that it localized in the cytoplasm [5]. The aim of this study was to investigate *NFXL1* at both the transcript and the protein levels.

## Methods

To detect the *NFXL1* transcript in various tissues, Amsbio cDNA human adult normal tissue major panels 1 and 2 (heart, brain, kidney, liver, lung, pancreas, spleen, skeletal muscle, and placenta) were used. RNA obtained from Clontech was used to examine differential expression in the brain. The samples consisted of RNA pooled from the brains of several adult donors (numbers vary by sample): whole brain, temporal lobe, frontal lobe, parietal lobe, cerebral cortex, and cerebellum. The QuantiTect Reverse Transcription kit (Qiagen) was used with 0.5 μg RNA to produce cDNA for use in a quantitative PCR (qPCR). cDNA for the brain regions was diluted 1:10 for use in qPCR on a Bio-Rad iQ5 machine. cDNA from the tissue panels was not diluted. The following TaqMan assays (Life Technologies) were used: Hs00541755_m1 (NFXL1) and Hs99999905_m1 (GAPDH, used as a housekeeping gene) with the standard TaqMan protocol. RNA-negative controls and reverse-transcriptase-negative controls were used. cDNA samples originating from RNA-positive samples were run as three replicates, and cycle-threshold values were averaged across all replicates for downstream analyses. Relative expression levels were estimated using the ∆∆*C*_T_ method [6] with samples normalized to the whole brain.

Immunofluorescence (IF) and western blot (WB) experiments were performed with HEK-293T cells grown in Dulbecco’s Modified Eagle’s Medium (Sigma) supplemented with 2 mM l-glutamine, 10 % fetal bovine serum, and 1 % penicillin/streptomycin. HEK cells naturally expressed *NFXL1* (detected using the TaqMan assay), and the presence of the endogenous protein was confirmed through western blotting. IF experiments were also performed with a neuronal cell line, SH-SY5Y. SH-SY5Y cells were grown in a medium consisting of 1:1 Minimum Essential Medium Eagle (Sigma) and Nutrient Mixture F-12 Ham (Sigma). The medium was supplemented with 2 mM l-glutamine, 1 % non-essential amino acids, 15 % fetal bovine serum, and 1 % penicillin/streptomycin. The cells were grown at 37 °C and 5 % CO_2_. The medium was changed every 1–3 days, and when the cells were at least 80 % confluent, the cell culture was split using trypsin/EDTA.

HEK cell RNA was extracted with RNeasy Mini kit (Qiagen). The presence in HEK cells of the transcripts of the HLA class II genes included in the study of HLA and SLI was assessed with TaqMan assays: HLA-DRB1 (Hs04192464_mH), HLA-DQA1 (Hs03007426_mH), and HLA-DQB1 (Hs03054971_m1). Cells were transfected with a plasmid containing the *NFXL1* cDNA and a Myc-DDK tag (RC216989, Origene) with 0.75 μg plasmid and 1 μl jetPRIME (Polyplus transfections) for IF for HEK cells (0.5 μg plasmid for SH-SY5Y cells) (24-well plate, using coverslips coated with poly-L-lysine) and 3 μg plasmid and 4 μl jetPRIME for WB (6-well plate). Control cells for WB were transfected with the empty vector (pCMV6-Entry Vector, Origene), while control cells for IF were not transfected. Cells were analyzed 24 h after the transfection medium had been changed (4 h after transfection). For IF, cells were fixed with cold methanol for 10 min and subjected to treatment with a blocking and permeabilization solution (0.1 % Triton X-100, 10 mg/ml (1 %) fish gelatin, 10 % normal goat serum in TBS). The primary antibodies were rabbit anti-NFXL1 A303-197A (Bethyl) and mouse anti-DDK TA50011-100 (Origene). The secondary antibodies were Alexa Fluor-conjugated antibodies anti-rabbit (594, red) and anti-mouse (488, green). The dilution factor for all antibodies was 1:500. Cells were also stained with DAPI (4',6-diamidino-2-phenylindole). The cells were stained for 1 h and washed with PBS five times after each staining for 10 min (three washes after DAPI staining). A Zeiss LSM 510 Meta Confocal Microscope was used in the IF experiments. For WB, cells were lysed on ice for 30 min using 50 mM Tris-HCl pH 7.8, 150 mM NaCl, 1 mM EDTA, 1 % Triton X-100, and 1× protease inhibitor cocktail (Roche). Protein concentration was measured with Pierce BCA Assay (Thermo Fisher). Electrophoresis and transfer were performed using the NuPAGE system (Invitrogen) following the manufacturer protocol for 7 % Tris-Acetate mini gels. The samples were heated at 70 °C for 10 min prior to being loaded onto the gel. Following the transfer, the PVDF membrane was blocked in 5 % skimmed milk dissolved in TBS Tween-20 0.1 % for at least 30 min. Blotting was performed with anti-NFXL1 A303-197A 1:2000 (in 1 % skimmed milk in TBS Tween-20) overnight at 4 °C. The secondary antibody was goat anti-rabbit IgG (H+L)-HRP conjugate (Bio-Rad) 1:5000 (1-h blotting at room temperature). After each blotting, the membrane was washed with TBS Tween-20 three times (10 min each). Amersham Biosciences ECL Plus kit was used. Pictures were taken with a Bio-Rad ChemiDoc MP imaging system.

## Results

The *NFXL1* transcript was detected (i.e., *C*_T_ values were obtained) in the heart, brain, kidney, liver, lung, pancreas, spleen, skeletal muscle, and placenta. A quantitative expression analysis across different tissues through PCR is problematic due to the fact that expression levels of housekeeping genes may differ across tissues, but the fact that *C*_T_ values were obtained for all tissues indicates that the transcript was present in all samples. A qPCR with cDNA from several brain regions associated with language was performed to assess the expression levels of *NFXL1* in those regions. As shown in Table 1, the cerebellum had the highest expression level. Additionally, a regular PCR and a gel electrophoresis were performed to confirm the presence of the transcript in samples from the various brain regions, and it was found in all of them (Additional file 1).

**Table 1:** Expression analysis results. Mean *C*_T_ values from qPCR for examined brain regions for *NFXL1* and *GAPDH*, standard deviations for measurements of three technical replicates, and expression levels relative to the whole brain

	Whole brain	Temporal lobe	Frontal lobe	Parietal lobe	Cerebral cortex	Cerebellum
*GAPDH* mean *C*_T_	24.06	21.84	21.93	21.63	21.96	22.14
*GAPDH* standard deviation	0.048	0.106	0.064	0.055	0.132	0.005
*NFXL1* mean *C*_T_	32.21	30.69	28.25	28.35	28.47	27.91
*NFXL1* standard deviation	0.267	0.04	0.213	0.157	0.193	0.244
*NFXL1* expression level in brain regions relative to the whole brain	1	0.62	3.56	2.69	3.12	5.21

**Fig. 1:**
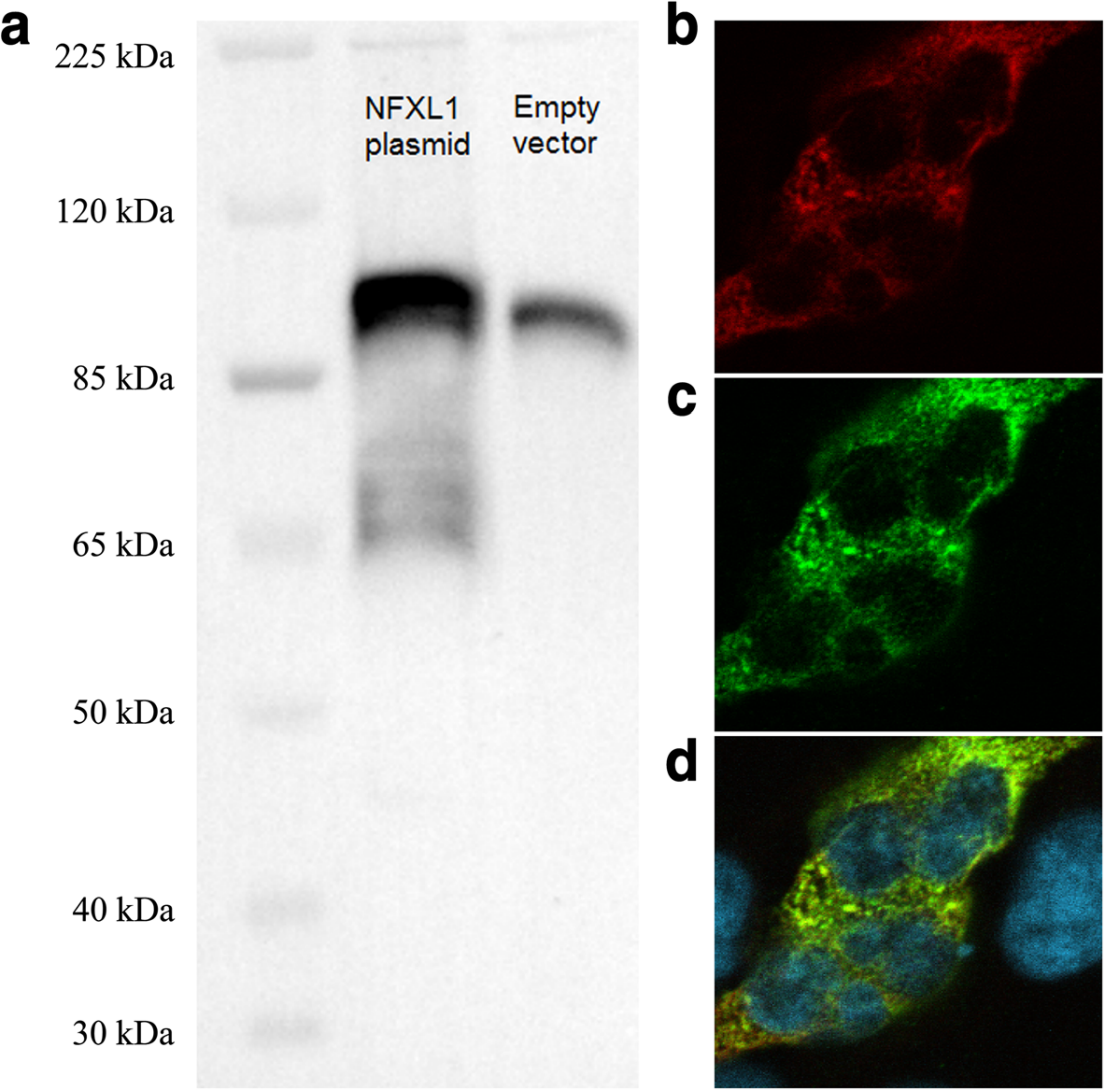
Western blot and immunofluorescence results for HEK cells. **a** Western blot image showing the overexpression of tagged NFXL1 in cells transfected with the NFXL1 plasmid compared to control cells. Samples of two micrograms of protein lysate were loaded onto the gel. Exposure time was 20 s. The protein ladder is superimposed with the WB image. The endogenous NFXL1 is expected to have a mass of 101 kDa. **b** Immunofluorescence of successfully transfected HEK cells showing overexpressed NFXL1 with the antibody for the endogenous protein. **c** Immunofluorescence of successfully transfected HEK cells showing overexpressed NFXL1 with the antibody for the DDK tag. **d** Superimposed images showing signals from both antibodies and a DAPI staining of the cell nuclei

**Fig. 2:**
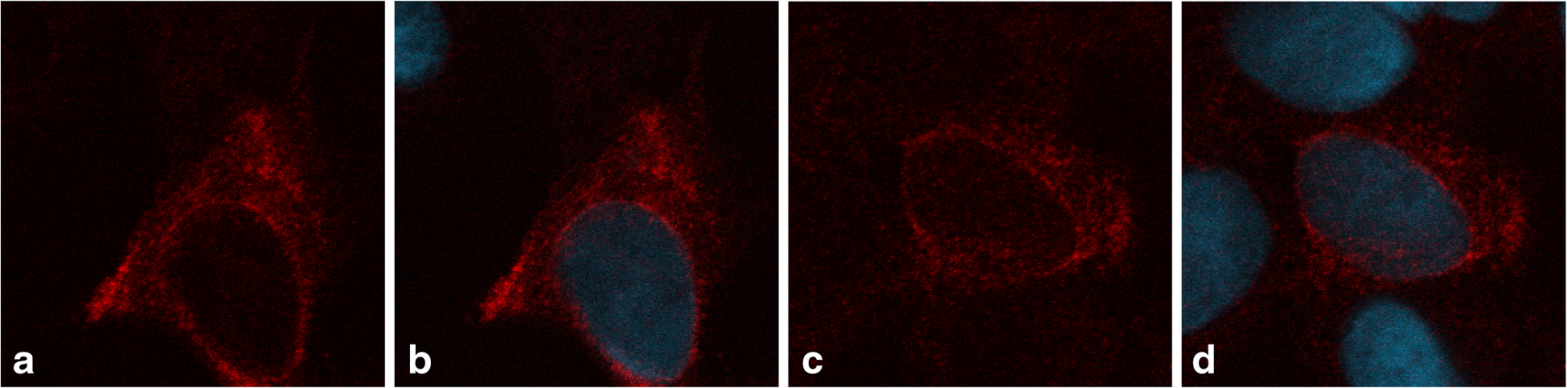
Immunofluorescence results for SH-SY5Y cells. **a** A representative cell from the transfected cell culture showing NFXL1 with the antibody for the endogenous protein. **b** A representative cell from the transfected cell culture showing NFXL1 with the antibody for the endogenous protein superimposed with the DAPI staining of the cell nuclei. **c** A representative cell from the control cell culture showing endogenous NFXL1 with the antibody for the endogenous protein. **d** A representative cell from the control cell culture showing endogenous NFXL1 with the antibody for the endogenous protein superimposed with the DAPI staining of the cell nuclei

WB analysis showed that the tagged protein was highly expressed and that it was slightly bigger than the endogenous protein (Fig. 1a). *C*_T_ values were obtained for all HLA class II genes studied using qPCR for HEK cell cDNA (*HLA-DRB1*, *HLA-DQA1*, and *HLA-DQB1*), suggesting that HEK cells naturally expressed putative NFXL1 targets. Two out of those three HLA class II genes (*HLA-DRB1* and *HLA-DQA1*) showed association with SLI [4]. HEK cells were therefore used to examine the cellular localization of the protein through IF. In HEK cells transfected with the *NFXL1* plasmid, the overexpressed protein was observed in the cytoplasm, where it could be detected with both the antibody for the endogenous protein and the antibody for the tag (Fig. 1b–d). Endogenous NFXL1 was not detected in control cells using the antibody for the endogenous protein in the IF experiments, possibly due to low levels of the endogenous protein compared to the background signal, although it was detected through WB (Fig. 1a). The IF experiment was performed again, with SH-SY5Y cells. No signal distinguishable from the background was detected with the antibody for the tag. However, a strong signal from the antibody for the endogenous protein was obtained for some cells in both the transfected and the control cultures. In those cases, the signal was not nuclear and appeared cytoplasmic, as in the case of the overexpressed protein in HEK cells (Fig. 2a–d).

## Discussion

Recently, mutations in *NFXL1* have been implicated in SLI [2]. The present study shows that *NFXL1* is expressed in many different types of tissues. While testing for relative expression levels across different tissues through qPCR is problematic due to differences in the expression levels of housekeeping genes across different tissues, *GAPDH* has been shown to have little variation in tissues belonging to the nervous system [7]. The results show that the *NFXL1* expression level in the cerebellum is more than five times higher than that in the whole brain. While the cerebellum is mostly thought of in the context of motor function, some studies have implicated it in language disorders: Silveri et al. reported on a patient with a right cerebellar infarction who developed problems with the morphological component of language [8]. Fabbro et al. reported on four patients with cerebellar lesions who had problems with morphology and syntax [9]. The high expression level in the cerebellum is in line with the high expression of *NFXL1* reported for the cerebellar cortex in the Allen Brain Atlas [10], with a maximum *Z*-score of 2.12945. Similarly, the highest reads per kilobase of transcript per million mapped reads (RPKM) values for *NFXL1* across all brain tissues as reported in the GTEx database [11] were those obtained for the cerebellar hemisphere and the cerebellum (median RPKM values of 3.916 and 3.906, respectively).

The IF experiments showed a strong cytoplasmic signal from the overexpressed NFXL1 protein in HEK cells and the endogenous protein in SH-SY5Y cells. In HEK cells, the localization could be checked with the antibody for the tag as well as the antibody for the endogenous protein at the same time (with antibodies from two different species). The results show that both antibodies provided the same signal (red and green in Fig. 1b, c, respectively), suggesting that both antibodies detected the same protein. It is possible that the tag influenced the localization of the protein. However, tagged proteins are routinely used in ChIP-Seq experiments to study nuclear proteins [12], and, in the case of the SH-SY5Y cells, some of the control cells showed cytoplasmic localization of endogenous NFXL1, i.e., when a signal was detected, it was cytoplasmic (Fig. 2c). These results are in line with a previous study, in which a small part of the *NFXL1* gene was cloned into a vector with a green fluorescent protein tag and transfected into HeLa cells. The resulting fusion protein localized in the cytoplasm [5]. In UniProt [13], the protein is annotated as nuclear and membranal, based on different predictions.

## Conclusions

This investigation is of a descriptive nature and was designed as a primary study of *NFXL1*. It provides experimental evidence that highlighted its high expression in a brain region that is relevant to language and experimentally confirmed its presence in a variety of tissues. The protein was not observed in the cell nucleus in examined cell lines, suggesting that, if it functions as a transcription factor, it may translocate to the nucleus under conditions not present in this study. Further functional studies are required in order to elucidate the involvement of *NFXL1* in SLI. The development of high-grade antibodies for NFXL1 and large-scale analyses of multiple cell lines in which NFXL1 might show nuclear localization would allow us to achieve a better understanding of its function and potential role in neural development.

### Availability of data and materials

The materials described in this study are available commercially. Reagents, kits, and RNA/cDNA samples were obtained from the companies mentioned in the main text.

## Additional file

Additional file 1: Amplification of a fragment of *NFXL1* cDNA in examined brain regions. Gel electrophoresis picture showing a fragment of *NFXL1* cDNA amplified by regular PCR, and the PCR protocol. (PDF 46.2 kb)

## Abbreviations

DAPI: 4',6-diamidino-2-phenylindole
IF: immunofluorescence
qPCR: quantitative PCR (polymerase chain reaction)
RPKM: reads per kilobase of transcript per million mapped reads
SLI: specific language impairment
WB: western blot
