# Supplementary material for: An investigation of NFXL1, a gene implicated in a study of specific language impairment
Source: J Neurodev Disord. 2016 Apr 5;8:13. doi: 10.1186/s11689-016-9146-9 (PMC4822306; doi:10.1186/s11689-016-9146-9)
Supplement: Additional file 1: — Amplification of a fragment of NFXL1 cDNA in examined brain regions. Gel electrophoresis picture showing a fragment of NFXL1 cDNA amplified by regular PCR, and the PCR protocol. (PDF 46.2 kb) [file 11689_2016_9146_MOESM1_ESM.pdf]

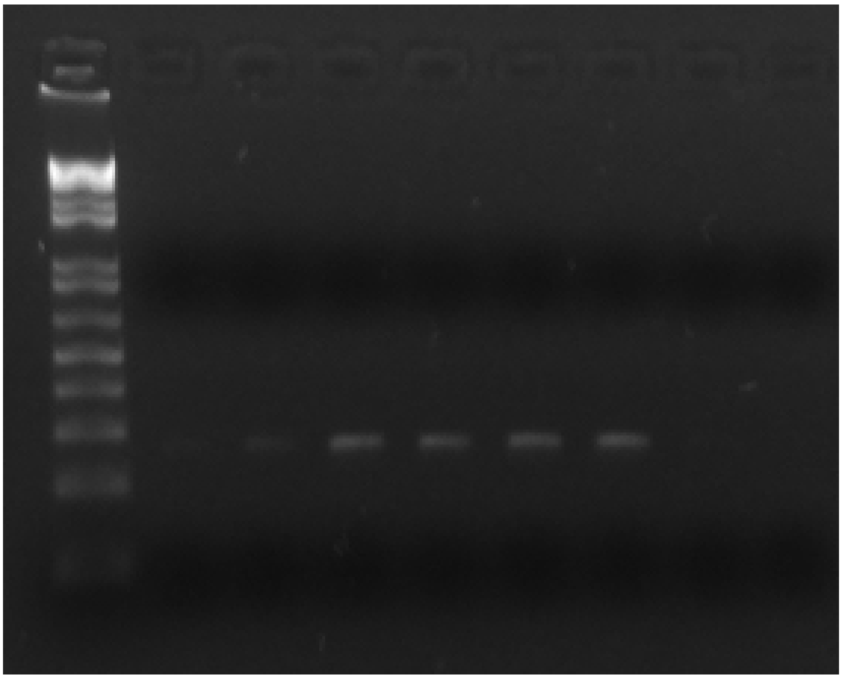

Gel picture showing a fragment of *NFXL1* cDNA amplified by regular PCR (primer sequences available upon request). Samples from left to right: 1 kb Plus DNA ladder (Invitrogen), whole brain, temporal lobe, frontal lobe, parietal lobe, cerebral cortex, cerebellum, colon, water control. A 2% agarose gel was used.

PCR protocol:

1. 95°C for 10 minutes
2. 95°C for 30 seconds
3. 55°C for 30 seconds
4. 72°C for 30 seconds
5. Steps 2-4×29
6. 72°C for 5 minutes

The PCR mix consisted of: 5 µl cDNA (diluted from RT-PCR reaction as detailed in the main text); 1 µl of the forward primer (10 µm); 1 µl of the reverse primer (10 µm); 5 µl of buffer (160 mM $(NH_4)_2SO_4$, 670 mM Tris-HCL, 0.1% stabiliser); 2.5 µl of $MgCl_2$ (50 mM); 1.25 µl of deoxyribonucleotides (dNTPs) (10 mM); 0.2 µl of 9:1 BIOTAQ (Bioline):Pfu (Thermo Scientific) (5 u/µl); 34.05 µl of MilliQ H_2O to a final volume of 50 µl.
